# “Could She/He Walk Out of the Hospital?”: Implementing AI Models for Recovery Prediction and Doctor-Patient Communication in Major Trauma

**DOI:** 10.3390/diagnostics15131582

**Published:** 2025-06-22

**Authors:** Li-Chin Cheng, Chung-Feng Liu, Chin-Choon Yeh

**Affiliations:** 1 Division of Colorectal Surgery, Department of Surgery, Chi Mei Medical Center, Tainan 710402, Taiwan; leproxy@gmail.com; 2 Division of Traumatology, Department of Surgery, Chi Mei Medical Center, Tainan 710402, Taiwan; 3 Department of Medical Research, Chi Mei Medical Center, Tainan 710402, Taiwan; chungfengliu@gmail.com; 4 Division of Plastic Surgery, Department of Surgery, Chi Mei Medical Center, Tainan 710402, Taiwan; 5 Department of Occupational Therapy, Shu-Zen Junior College of Medicine and Management, Kaohsiung 821004, Taiwan; 6 Department of Physical Therapy, Shu-Zen Junior College of Medicine and Management, Kaohsiung 821004, Taiwan

**Keywords:** major trauma patient, prognosis, recovery, mortality, artificial intelligence, machine learning, hospital information systems

## Abstract

**Background and Objectives**: Major trauma ranks among the leading causes of mortality and handicap in both developing and developed countries, consuming substantial healthcare resources. Its unpredictable nature and diverse clinical presentations often lead to rapid and challenging-to-predict changes in patient conditions. An increasing number of models have been developed to address this challenge. Given our access to extensive and relatively comprehensive data, we seek assistance in making a meaningful contribution to this topic. This study aims to leverage artificial intelligence (AI)/machine learning (ML) to forecast potential adverse effects in major trauma patients. **Methods**: This retrospective analysis considered major trauma patient admitted to Chi Mei Medical Center from 1 January 2010 to 31 December 2019. **Results**: A total of 5521 major trauma patients were analyzed. Among five AI models tested, XGBoost showed the best performance (AUC 0.748), outperforming traditional clinical scores such as ISS and GCS. The model was deployed as a web-based application integrated into the hospital information system. Preliminary clinical use demonstrated improved efficiency, interpretability through SHAP analysis, and positive user feedback from healthcare professionals. **Conclusions**: This study presents a predictive model for estimating recovery probabilities in severe burn patients, effectively integrated into the hospital information system (HIS) without complex computations. Clinical use has shown improved efficiency and quality. Future efforts will expand predictions to include complications and treatment outcomes, aiming for broader applications as technology advances.

## 1. Introduction

Major trauma arouses widespread concern among healthcare professionals and the general public across all age groups worldwide [1,2,3]. The diversity and variability of major trauma also pose numerous challenges for clinical practitioners. Predicting trauma outcomes aids clinicians in prioritizing patients and delivering timely, effective treatment. Various scoring systems have been employed to forecast prognosis and mortality in these cases [4,5]. The Injury Severity Score (ISS), New Injury Severity Score (NISS), Revised Trauma Score (RTS), and Trauma and Injury Severity Score (TRISS) stand as widely employed scoring systems in clinical practice [6]. In addition, the GCS scoring system is also used to assess the prognosis of the nervous system [7].

An evolving body of literature underscores the significance of personalized diagnostic, prognostic, and therapeutic tools in steering the cost-effective care of patients post-traumatic injury [8].

Nevertheless, the aforementioned scoring methods offer only constrained clinical predictive insights and may not adeptly address the dynamic nature of evolving medical conditions and the varied consultation needs expressed by patients and their families. This can result in a communication and information gap between healthcare providers and patients or their family members. The issues they are most concerned about, besides the severity of the injuries, are the prospects of recovery and discharge in the future.

In recent years, trends in big data, AI, and machine learning have been flourishing, and many scholars have applied them to trauma patients [8,9,10,11,12]. Indeed, AI applications in clinical medicine, such as complication prediction, have already yielded rich and exciting results. For example, in fields like surgery [13,14,15], internal medicine [16,17], and obstetrics [18], there have been excellent papers and studies published. As clinical physicians, this is something we are pleased to see. However, in the practical application to local patients, we have encountered an awkward situation of mismatched data and results that are not applicable.

Given this situation, there is an important and essential need to create more real-time and high-quality trauma prediction tools that align with the demands of modern precision and personalized medicine. We aim to develop a predictive model that can be integrated with the Hospital Information System (HIS) and combined with a visual interface to facilitate communication with patients and their families. The contribution of the recovery prediction is to provide early reminders about the need for ongoing care and rehabilitation, helping patients and families prepare in advance. Consequently, our research endeavors to build a predictive model for major trauma patients, especially on the focus of recovery possibilities, employing AI/ML methodologies. This will be based on an extensive database of trauma patients from a Taiwanese trauma center.

## 2. Materials and Methods

### 2.1. Study Design, Setting, and Samples

**Figure 1:**
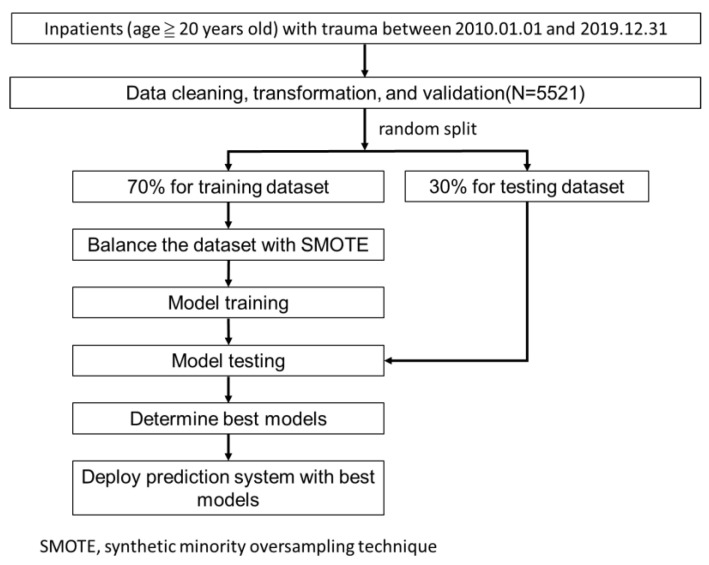
Research flow.

All inpatients with major trauma from 2010 to 2019 were included. Overall, 5521 cleaned cases were used in the study. Figure 1 illustrates our research flow.

The study was approved by the Institutional Review Board of the Chi Mei Medical Center (IRB Serial No.: 11206-015; 12 July 2023). All methods were carried out following relevant guidelines and regulations. Informed consent from patients was waived due to the retrospective nature of the study.

### 2.2. Feature and Outcome Variables

The outcome variable is defined as “whether a major trauma patient achieves recovery within 2 months after the injury”. This refers to patients who are successfully discharged home, excluding those transferred to nursing facilities or placed in long-term care.

11 feature variables, based on literature review and clinical experience, include:

(1) Demographics: gender (male or female), age
(2) Arrival Information: mode of arrival (BIBA (Brought in by Ambulance) or PAT (Privately Arranged Transport))
(3) Injury Severity: GCS (Glasgow Coma Scale), ISS (Injury Severity Score)
(4) Clinical Intervention: endotracheal intubation
(5) Vital Signs: SBP (Systolic Blood Pressure), DBP (Diastolic Blood Pressure), RR (Respiratory Rate), HR (Heart Rate)
(6) Hospital Days (Length of Stay)

Since the hospital length of stay is not known at the time of patient admission, the prediction model was actually developed using the remaining 10 variables.

### 2.3. Model Building and Performance Evaluation

All available variables were included in the predictive models without feature selection preprocessing to maximize overall model performance. The data were randomly divided into a training set (70%) and a testing set (30%) using stratified sampling. To address the class imbalance caused by the relatively small proportion of non-recovery cases in the training set, we employed a synthetic minority oversampling technique (SMOTE) [19]. Five distinct machine learning algorithms were used to model each outcome: (1) logistic regression, (2) random forest, (3) LightGBM, (4) XGBoost, and (5) MLP. A grid search with five-fold cross-validation was conducted on the training dataset to identify the optimal configuration for each model. Finally, the performance of the models was evaluated on the testing dataset using accuracy, sensitivity, specificity, and the area under the receiver operating characteristic curve (AUC) as performance metrics.

### 2.4. Implementation and Deployment of the Best Models

The model that achieved the highest AUC value was selected as the best one and then utilized to develop a web-based prediction application, which was deployed in clinical settings to assist physicians’ decision-making. The application was implemented using Microsoft Visual Studio^®^ (version 17.7).

## 3. Results

### 3.1. Demographics

From 1 January 2010 to 31 December 2019, a total of 5521 major trauma adult patients (≥20 years old) were enrolled in the study. Table 1 presents the demographic and clinical characteristics of major trauma patients, categorized by recovery and non-recovery outcomes. In total for recovery cases, the mean age was 54.14, and most patients were males (62.54%); approximately 84.54% of patients were admitted via BIBA, while around 15.46% arrived through PAT; the mean length of hospital stays is 14.35 days.

### 3.2. Machine-Learning Modeling Results

Overall, 70% of the data were randomly split for model training, and 30% for model evaluating (testing). Table 2 compares the classification performance of five algorithms—XGBoost, Logistic Regression, Random Forest, LightGBM, and MLP—by reporting their respective accuracy, sensitivity, specificity, and AUC. XGBoost achieves the highest accuracy (0.716) and the highest AUC (0.748), suggesting that it provides a strong balance between true-positive recognition and overall discrimination capability. Although Random Forest and XGBoost share the highest sensitivity (0.751), XGBoost maintains a notably higher specificity (0.627) compared to the other models, indicating its superior ability to correctly identify negative cases. Logistic Regression, Random Forest, and MLP each yield comparable accuracies (ranging from 0.708 to 0.709) and similar sensitivities (0.750–0.751), but their specificity values (0.601–0.603) and AUCs (0.731–0.743) do not surpass those of XGBoost. LightGBM, though offering a sensitivity of 0.750, shows a slightly lower specificity of 0.591 and an AUC of 0.738. Overall, XGBoost emerges as the most robust performer, combining high accuracy, competitive sensitivity, and leading specificity and AUC.

### 3.3. Interpreting the Feature Importance

**Figure 2:**
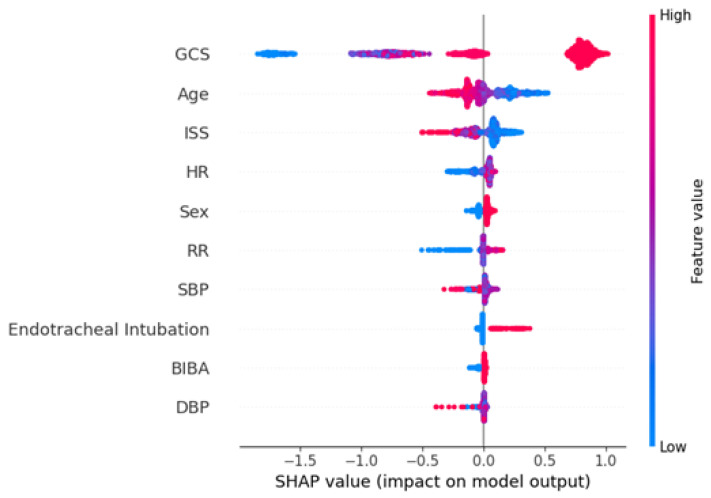
SHAP plot for the best model of recovery (XGBoost).

Furthermore, for a better interpretation of how each feature contributes to the associated outcome, we performed a SHAP (SHapley Additive exPlanations) analysis for each best AI model. In the SHAP global explanation plot, dots in red and blue indicate higher and lower feature values, respectively. A dot distribution to the left of the horizontal axis point 0 represents a negative correlation to the outcome, while a distribution to the right represents a positive correlation. The *y*-axis indicates the feature name, in order of importance from the top to the bottom of the plot. The wider the dots of the feature distributed are, the greater is the influence of the feature on the outcome. Figure 2 depicts the global explanations of the best model using a SHAP plot.

From an overall perspective, GCS exerts a notably strong influence on outcome prediction, as reflected by its wide range of SHAP values. Higher GCS values (indicated in red) tend to shift the predicted outcome to one side of the axis, whereas lower GCS values (in blue) push it in the opposite direction, highlighting the pivotal role of coma scores within this clinical context. Age and ISS also exhibit distributions spanning both negative and positive regions, implying that their specific values and interactions with other features may either increase or decrease the likelihood of adverse outcomes. For example, higher ISS values (in red) typically drive the prediction toward one side, suggesting that more severe injuries are associated with a lower probability of recovery and discharge.

In summary, the SHAP plot indicates that GCS, age, and ISS are the three most influential predictors of the recovery outcome. Vital signs and the mode of transport also contribute moderately to the prediction results. Specifically, higher GCS scores, younger age, lower ISS scores, the presence of endotracheal intubation, male gender, and patients transported by ambulance are associated with higher recovery rates. Conversely, patients with lower heart rates and respiratory rates are less likely to recover. The finding that males are more likely to recover may be due to the generally younger average age of male patients, which increases their likelihood of recovery. This comprehensive analysis of feature importance helps clinicians and researchers better understand how each variable impacts the model’s predictions, thereby enhancing its interpretability and clinical applicability.

### 3.4. Performance Comparison of AI Models

Based on the results in Table 2, XGBoost was selected for further comparisons with well-defined clinical scores due to its best performance across all metrics. As shown in Table 3, the AI prediction model (XGBoost) performs the best in predicting the outcomes of major trauma patients, with an accuracy of 0.716, sensitivity of 0.751, specificity of 0.627, and an AUC of 0.748. This indicates that the XGBoost model achieves a good balance between correctly identifying positive samples (sensitivity) and distinguishing negative samples (specificity), with its higher AUC value demonstrating stronger overall predictive capability.

In contrast, the Injury Severity Score (ISS) has an accuracy of 0.713, sensitivity of 0.771, specificity of 0.567, and an AUC of 0.689. Compared to the XGBoost model, its AUC is significantly lower. The higher sensitivity of the ISS means it can better identify high-risk patients, but its lower specificity suggests that it may misclassify some negative cases as positive (false positives).

The Glasgow Coma Scale (GCS) shows a lower predictive performance, with an accuracy of 0.663, sensitivity of 0.657, specificity of 0.678, and an AUC of 0.719. Compared to XGBoost and ISS, the prediction accuracy and AUC of the GCS are also significantly lower, indicating its less effective ability to predict the outcomes of major trauma patients.

Overall, the XGBoost model outperforms both the ISS and GCS across all metrics, including accuracy, sensitivity, specificity, and AUC, providing more accurate predictions for clinical decision-making.

### 3.5. Development, Deployment, and Preliminary User Evaluation of the Prediction System

**Figure 3:**
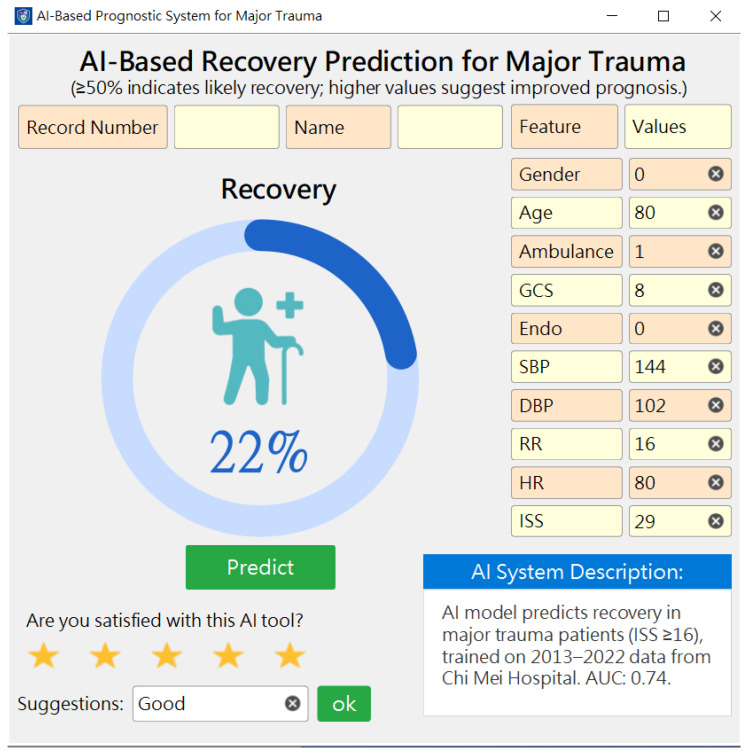

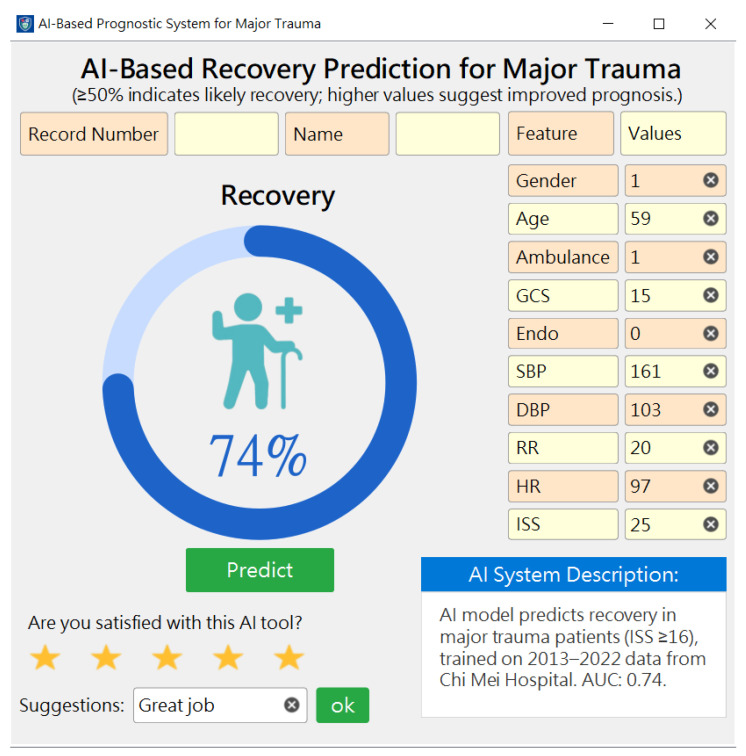
Two snapshots of the AI prediction system with the best model.

To clarify the feasibility and acceptance of our AI models, we developed an AI recovery prediction system based on the best model and deployed it for trauma patients to assist physicians in making decisions. Figure 3 shows a snapshot of the AI system (a probability ≥50% indicates a high probability of a recovery outcome). The model was built using Python (v3.9.1) programming language, and the web-based prediction system was developed using an MS Visual Studio^®^ with VB (v17.7).

We then demonstrated the AI system to the pilot users (three specialist nurse practitioners, one intensive care unit (ICU) physician, and one trauma specialist physician) and received positive feedback (mean satisfaction ≥4.0 on a 5-point scaled question). They viewed it as a useful tool for the timely identification of high-recovery probability patients. Based on the probability displayed in the system, healthcare staff can consider appropriate treatment plans to optimize medical resource utilization and effectively communicate with patients and their families, serving as a suitable shared decision-making (SDM) tool. This approach significantly improves the quality and efficiency of trauma patient care.

## 4. Discussion

In our study, XGBoost stands out as the most robust performer, offering high accuracy, strong sensitivity, and leading specificity and AUC. Next, we generated the SHAP plot for the top-performing recovery model (Figure 2). We found that a higher GCS, lower ISS, and younger age all predict better recovery outcomes, which aligns with general expectations and previous studies [20].

Vital signs and modes of transport also play a moderate role in prediction. Specifically, endotracheal intubation, male gender, and transport by ambulance are associated with higher recovery rates. These findings highlight the importance of early emergency care and the chain of survival [21,22].

The higher recovery likelihood in males may be attributed to their generally younger average age, which enhances their chances of recovery [23].

According to Table 3, our predictive model (XGBoost) outperforms GCS and ISS in both accuracy and specificity. This advantage likely stems from incorporating additional parameters, such as age and vital signs upon arrival, along with accounting for the quality of initial emergency care.

Furthermore, regarding the user experience of healthcare professionals, we obtained encouraging results from the satisfaction survey. Users believed that our system helps accelerate the prediction of medical conditions. We believe that this system is helpful in integrating a variety of meaningful information to make rapid preliminary judgments.

Next, we conducted a thorough review of several relevant and comparable studies. In fact, the application of information technology in the field of trauma medicine began quite early. As far back as 1988, Clarke et al. [24] developed a computerized decision support system designed to assist ATLS-trained surgeons in the initial definitive management of patients with penetrating abdominal injuries immediately following resuscitation and stabilization. Over time, in the early 21st century, Becalick and Coats introduced predictive models incorporating nonlinear methods to complement the limitations of purely linear approaches, such as the Injury Severity Score (ISS) [25].

Over the past three decades, the performance of computer hardware and software has advanced dramatically. Various AI-based image processing and diagnostic systems have been applied across multiple areas of trauma care. Exciting research has been published across various fields, including knee trauma [26], facial trauma [27], pediatric head trauma [28], and chest trauma [29,30].

And in the diagnosis and treatment of trauma in various organ systems, researchers’ studies have been emerging like bamboo shoots after a spring rain. Keller et al. examined the potential benefit of artificial intelligence regarding clinical decision-making in the treatment of wrist trauma patients [31]. Peng et al. focused their research on traumatic hemorrhage [32], while Lyu et al. introduced applications related to spinal cord trauma [33]. Additionally, Galwankar discussed the use of artificial intelligence in managing blunt chest trauma [34].

Moreover, we conducted a comprehensive comparison of our study with other similar research endeavors, which we have succinctly summarized in Table 4.

Based on the above comparison, each study utilized appropriate data and methods to predict the condition of patients with major trauma, achieving exciting results. Lee et al. [35] and Sun [36] conducted impressive investigations into trauma-related mortality and complications.

However, during the process of searching relevant literature, we found that researchers worldwide have rarely developed predictive models for the recovery probability of major trauma patients. We utilized a large number of patients and extracted useful data to develop this predictive model, which was practically applied in clinical work and integrated with the hospital’s medical record system. From the perspective of supporting clinical decision-making, a model with an AUC greater than 0.7 is considered to have good reference value. Additionally, our prediction tool is primarily designed to assist in decision-making and to facilitate communication between healthcare providers and patients. In clinical practice, when the predicted probability of recovery is less than 50%, medical staff will inform the family members to make early preparations, such as consulting long-term care facilities or arranging rehabilitation plans. The feedback from internal staff questionnaires was encouraging, primarily because it helped improve communication between physicians and patients’ families, thereby enhancing overall service satisfaction.

In our study, recovery is defined as being successfully discharged home, excluding those transferred to nursing facilities or placed in long-term care. However, we were unable to accurately define the patients’ ability to perform daily activities independently after being discharged home, which is a significant limitation of this study. Therefore, we will next attempt to further track the patient’s recovery after a successful discharge and return home. This will be conducted using objective scoring methods, such as the ADL score, in order to better reflect the patient’s functional status and vocational capabilities.

Secondly, although the database built by our department contains a large number of patients and extensive information, it still cannot cover all the necessary or meaningful data. This limitation prevented our AI predictive model from achieving optimal performance. Fortunately, we are pleased to find that the model received positive feedback in practical applications, which can be considered the greatest clinical significance of this study.

Furthermore, when using our institution’s self-constructed database, data integration does not pose a significant issue. However, to conduct multi-center validation, it is necessary for all collaborating institutions to jointly establish a standardized and interoperable database. This would allow for scaling up the dataset and enhancing its value for validation purposes. This initiative is also part of an ongoing plan by the government authorities.

In terms of future prospects, we first aim to enhance our data extraction techniques to obtain more accurate and comprehensive meaningful data. We also plan to improve our experimental design to achieve higher predictive accuracy. Of course, these results must be repeatedly validated through clinical practice, with the hope of bringing greater benefits to healthcare professionals and patients.

## 5. Conclusions

This study aims to provide a predictive model for estimating the recovery probability of patients with major trauma, to assist medical professionals in making informed prognostic assessments and responding to the concerns of anxious family members. Furthermore, we successfully developed and smoothly integrated a web-based predictive application into the existing hospital information system (HIS) without the need for complex computational processes. This model has been practically utilized by clinical healthcare professionals, and it has been found to significantly enhance work efficiency and quality.

Next, we will attempt to predict various outcomes, including a range of complications as well as positive and negative treatment results. We believe that with the continued advancement of information technology in the future, we will be able to develop better predictive models to assist in more aspects.

## Figures and Tables

**Table 1:** The demographic and clinical characteristics of recovery and non-recovery.

Variable	Non-Recovery *n* = 1562	Recovery *n* = 3959	*p*-Value
Age (years), mean ± SD	59.11 ± 19.26	54.14 ± 20.09	<0.001
Gender, *n* (%)			
Female	498 (31.88%)	1483 (37.46%)	<0.001
Male	1064 (68.12%)	2476 (62.54%)	
Mode of Arrival (to the ED), *n* (%)			
BIBA	1429 (91.49%)	3347 (84.54%)	<0.001
PAT	133 (8.52%)	612 (15.46%)	
GCS, mean ± SD	10.24 ± 4.78	13.70 ± 2.66	<0.001
ISS, mean ± SD	26.02 ± 12.15	20.83 ± 6.52	<0.001
Endotracheal Intubation, *n* (%)	213 (13.63%)	139 (3.51%)	<0.001
SBP, mean ± SD	141.67 ± 47.86	146.63 ± 34.80	<0.001
DBP, mean ± SD	82.81 ± 26.33	86.26 ± 18.51	<0.001
RR, mean ± SD	16.85 ± 5.52	17.77 ± 2.89	<0.001
HR, mean ± SD	87.99 ± 27.01	88.80 ± 18.10	0.276
Hospital Days, mean ± SD	22.92 ± 24.76	14.35 ± 14.92	<0.001

Notes. ED = Emergency Department, BIBA = brought in by ambulance, PAT = privately arranged transport, GCS = Glasgow Coma Scale, ISS = injury severity score, SBP = systolic blood pressure, DBP = diastolic blood pressure, RR = respiratory rate, HR = heart rate.

**Table 2:** Recovery model performance.

Algorithm	Accuracy	Sensitivity	Specificity	AUC
XGBoost	0.716	0.751	0.627	0.748
Random Forest	0.709	0.751	0.603	0.743
LightGBM	0.705	0.750	0.591	0.738
Logistic Regression	0.708	0.750	0.601	0.732
MLP	0.709	0.750	0.603	0.731

**Table 3:** Comparison of the prediction accuracy of AI model, Injury Severity Score and Glasgow Coma Scale in predicting outcomes of patients with major trauma.

Outcomes and Predictive Models	Accuracy	Sensitivity	Specificity	AUC	*p*-Value
AI prediction (XGBoost)	0.716	0.751	0.627	0.748	
Injury Severity Score	0.713	0.771	0.567	0.689	<0.001
Glasgow Coma Scale	0.663	0.657	0.678	0.719	<0.001

Note: the *p*-value resulting from the DeLong test was obtained to assess the statistical significance of the performance difference between the two models.

**Table 4:** A comparison with similar studies.

Study	This Study	[35]	[36]
Patient number	5521	921	961
Types of Patient Origin	Adult inpatient with major trauma (database built by our trauma center)	Korean National Emergency Department Information System (NEDIS) data set	Major trauma at their hospital + a database in the United States
Outcome	Recovery (whether a major trauma patient achieves recovery within 2 months after the injury)	In-hospital mortality	Sepsis
Study method	Five machine leaning methods	Various machine learning models with an ensemble approach via 5-fold cross-validation	Six machine leaning methods
Real world implementation	Yes A predictive application with AI models was implemented and integrated into the existing HIS	Yes Deployed their final AI model on a public website	Yes Medical providers can access the application by clicking on the URL provided in the study
Input data	10 patient demographic features: including ISS, GCS, vital signs, BIBA or PAT, endotracheal intubation	10 patient demographic features: including Korean Triage and Acuity Scale, GCS, vital signs	12 patient demographic features: including ISS, GCS, vital signs, smoking, trauma part, lab. data
Testing results (AUC)	Recovery (0.731–0.748)	In-hospital mortality (0.7675)	Sepsis (0.787–0.932)
Year	2025	2023	2025

## Data Availability

The dataset used for this study is available on request from the corresponding author.

## References

[1] Castelao M., Lopes G., Vieira M. (2023). Epidemiology of major paediatric trauma in a European Country—Trends of a decade. BMC Pediatr..

[2] Farrow L., Diffley T., Gordon M.W.G., Khan A., Capek E., Anand A., Paton M., Myint P.K. (2023). Epidemiology of major trauma in older adults within Scotland: A national perspective from the Scottish Trauma Audit Group (STAG). Injury.

[3] Montoya L., Kool B., Dicker B., Davie G. (2022). Epidemiology of major trauma in New Zealand: A systematic review. N. Z. Med. J..

[4] Alam A., Gupta A., Gupta N., Yelamanchi R., Bansal L., Durga C. (2021). Evaluation of ISS, RTS, CASS and TRISS scoring systems for predicting outcomes of blunt trauma abdomen. Pol. Przegl. Chir..

[5] Bogner V., Brumann M., Kusmenkov T., Kanz K.G., Wierer M., Berger F., Mutschler W. (2016). [Retrospective computation of the ISS in multiple trauma patients: Potential pitfalls and limitations of findings in full body CT scans]. Unfallchirurg.

[6] Hoke M.H., Usul E., Ozkan S. (2021). Comparison of Trauma Severity Scores (ISS, NISS, RTS, BIG Score, and TRISS) in Multiple Trauma Patients. J. Trauma Nurs..

[7] Mahajan C., Sengupta D., Kapoor I., Prabhakar H., Kumar V., Purohit S., Priya V., Srivastava S., Thakur D., Karnik H. (2023). Evaluation of the GCS-Pupils Score for PrOgnosis in trauMatic brAin injury- The COMA Study. Brain Inj..

[8] Moris D., Henao R., Hensman H., Stempora L., Chasse S., Schobel S., Dente C.J., Kirk A.D., Elster E. (2022). Multidimensional machine learning models predicting outcomes after trauma. Surgery.

[9] Abujaber A., Fadlalla A., Gammoh D., Abdelrahman H., Mollazehi M., El-Menyar A. (2020). Using trauma registry data to predict prolonged mechanical ventilation in patients with traumatic brain injury: Machine learning approach. PLoS ONE.

[10] Abujaber A., Fadlalla A., Gammoh D., Abdelrahman H., Mollazehi M., El-Menyar A. (2020). Prediction of in-hospital mortality in patients with post traumatic brain injury using National Trauma Registry and Machine Learning Approach. Scand. J. Trauma Resusc. Emerg. Med..

[11] Cardosi J.D., Shen H., Groner J.I., Armstrong M., Xiang H. (2021). Machine learning for outcome predictions of patients with trauma during emergency department care. BMJ Health Care Inform..

[12] Lee K.C., Hsu C.C., Lin T.C., Chiang H.F., Horng G.J., Chen K.T. (2022). Prediction of Prognosis in Patients with Trauma by Using Machine Learning. Medicina.

[13] Zhu Y., Du M., Li P., Lu H., Li A., Xu S. (2025). Prediction models for the complication incidence and survival rate of dental implants-a systematic review and critical appraisal. Int. J. Implant. Dent..

[14] van de Beld J.J., Crull D., Mikhal J., Geerdink J., Veldhuis A., Poel M., Kouwenhoven E.A. (2024). Complication Prediction after Esophagectomy with Machine Learning. Diagnostics.

[15] Florquin R., Florquin R., Schmartz D., Dony P., Briganti G. (2024). Pediatric cardiac surgery: Machine learning models for postoperative complication prediction. J. Anesth..

[16] Shiren Y., Jiangnan Y., Xinhua Y., Xinye N. (2024). Interpretable prediction model for assessing diabetes complication risks in Chinese sufferers. Diabetes Res. Clin. Pract..

[17] Bosnjic J. (2024). Prediction of pulmonary embolism and its complication in diabetes mellitus type 2: A 5-year retrospective study. Med. Glas..

[18] O’Connor S., Tilston G., Jones O., Sharma A., Ormesher L., Quinn B., Wilson A., Myers J., Peek N., Palin V. (2024). Acceptability of data linkage to identify women at risk of postnatal complication for the development of digital risk prediction tools and interventions to better optimise postnatal care, a qualitative descriptive study design. BMC Med..

[19] Chawla N.V., Bowyer K.W., Hall L.O., Kegelmeyer W.P. (2002). SMOTE: Synthetic minority over-sampling technique. J. Artif. Intell. Res..

[20] Maye H., Waqar M., Colombo F., Lekka E. (2023). External validation of the GCS-Pupils Score as an outcome predictor after traumatic brain injury in adults: A single-center experience. Acta Neurochir..

[21] Bakke H.K., Wisborg T. (2017). The trauma chain of survival—Each link is equally important (but some links are more equal than others). Injury.

[22] Soreide K. (2012). Strengthening the trauma chain of survival. Br. J. Surg..

[23] Haering S., Schulze L., Geiling A., Meyer C., Klusmann H., Schumacher S., Knaevelsrud C., Engel S. (2024). Higher risk-less data: A systematic review and meta-analysis on the role of sex and gender in trauma research. J. Psychopathol. Clin. Sci..

[24] Clarke J.R., Cebula D.P., Webber B.L. (1988). Artificial intelligence: A computerized decision aid for trauma. J. Trauma.

[25] Becalick D.C., Coats T.J. (2001). Comparison of artificial intelligence techniques with UKTRISS for estimating probability of survival after trauma. UK Trauma and Injury Severity Score. J. Trauma.

[26] Hong G., Zhang L., Kong X., Herbertl L. (2021). Artificial Intelligence Image-Assisted Knee Ligament Trauma Repair Efficacy Analysis and Postoperative Femoral Nerve Block Analgesia Effect Research. World Neurosurg..

[27] Pham T.D., Holmes S.B., Coulthard P. (2023). A review on artificial intelligence for the diagnosis of fractures in facial trauma imaging. Front. Artif. Intell..

[28] Azad Z., Aiman U., Shaheen S. (2024). Artificial intelligence in paediatric head trauma: Enhancing diagnostic accuracy for skull fractures and brain haemorrhages. Neurosurg. Rev..

[29] Kaike L., Castro-Zunti R., Ko S.B., Jin G.Y. (2024). [Diagnosis of Rib Fracture Using Artificial Intelligence on Chest CT Images of Patients with Chest Trauma]. J. Korean Soc. Radiol..

[30] Zhao T., Meng X., Wang Z., Hu Y., Fan H., Han J., Zhu N., Niu F. (2024). Diagnostic evaluation of blunt chest trauma by imaging-based application of artificial intelligence. Am. J. Emerg. Med..

[31] Keller M., Rohner M., Honigmann P. (2024). The potential benefit of artificial intelligence regarding clinical decision-making in the treatment of wrist trauma patients. J. Orthop. Surg. Res..

[32] Peng H.T., Siddiqui M.M., Rhind S.G., Zhang J., da Luz L.T., Beckett A. (2023). Artificial intelligence and machine learning for hemorrhagic trauma care. Mil. Med. Res..

[33] Lyu S.Q., Yang L., Chen L. (2024). [The application of artificial intelligence in prehospital treatment of spinal cord trauma]. Zhonghua Yi Xue Za Zhi.

[34] Galwankar S., Szarpak L., Cander B., Maj B., Pruc M. (2025). The use of artificial intelligence in blunt chest trauma. Am. J. Emerg. Med..

[35] Lee S., Kang W.S., Kim D.W., Seo S.H., Kim J., Jeong S.T., Yon D.K., Lee J. (2023). An Artificial Intelligence Model for Predicting Trauma Mortality Among Emergency Department Patients in South Korea: Retrospective Cohort Study. J. Med. Internet Res..

[36] Sun B., Lei M., Wang L., Wang X., Li X., Mao Z., Kang H., Liu H., Sun S., Zhou F. (2025). Prediction of sepsis among patients with major trauma using artificial intelligence: A multicenter validated cohort study. Int. J. Surg..

